# A Comprehensive Analysis of Clinical Presentations, Laboratory Findings, and Etiologies of Pancytopenia: A Tertiary Care Experience

**DOI:** 10.7759/cureus.73148

**Published:** 2024-11-06

**Authors:** Waqas Fazal, Suleman Khan, Redaina Akhtar, Shayan Aziz Khattak, Muhammad Ali, Musa Kakakhel, Muhammad Numan Saleem, Obaid Ullah, Sumira Abbas, Faraz Ahmed Jamali

**Affiliations:** 1 Internal Medicine, Hayatabad Medical Complex Peshawar, Peshawar, PAK; 2 Oncology, University College of London Hospitals, London, GBR; 3 Internal Medicine, Khyber Teaching Hospital Peshawar, Peshawar, PAK; 4 Medicine, Sunderland Royal Hospital South Tyneside and Sunderland NHS Foundation Trust (STSFT), Sunderland, GBR; 5 Pathology, Kuwait Teaching Hospital, Peshawar, PAK; 6 Ear, Nose, and Throat, Doncaster Royal Infirmary, Doncaster, GBR

**Keywords:** bone marrow, causes of pancytopenia, laboratory findings of pancytopenia, megaloblastic anemia, pancytopenia

## Abstract

Background

Pancytopenia, while a common manifestation of a multitude of diseases, remains a relatively lesser-researched topic, especially in developing countries. Its management depends largely on identifying the etiology, which can range from simple infections to more sinister causes like leukemia. This study aims to investigate the clinical presentations, hematological findings, and etiologies of pancytopenia in a developing country.

Methods

A cross-sectional study was conducted at Hayatabad Medical Complex, Peshawar, and included 106 patients who were diagnosed with pancytopenia. Thorough demographic details, histories, clinical examinations, laboratory investigations, bone marrow biopsies, and final diagnoses were recorded and analyzed using statistical tools.

Results

Pancytopenia was most common in the age group 11-30 years with a male-to-female ratio of 1.4:1. Infections were the leading etiology (17.9%), followed by megaloblastic anemia (17%), hypersplenism (16%), and malignancy (15.1%). Among infections, enteric fever was the most frequently observed cause. The most common presentation was with signs and symptoms of anemia, followed by infections and thrombocytopenia. The most common blood smear finding case-wise was a combination of anisocytosis, microcytosis, and target cells. In patients who underwent bone marrow biopsy, the commonest finding (34%) was suggestive of malignancy with hypercellularity and abnormal cells. Aplastic anemia, hypersplenism, and malignancy were associated with a retic count of <1.5%. Platelet counts of less than 50,000 were associated with the presence of signs and symptoms of thrombocytopenia.

Conclusion

Pancytopenia can be a presenting feature of a reversible condition like underlying infection and megaloblastic anemia, which contribute a major portion of its etiologies. Early diagnosis and treatment can reverse pancytopenia and prevent over-investigation.

## Introduction

Pancytopenia is a decrease in the number of cells across all cell lines (i.e., red blood cells, white blood cells, and platelets) in blood. It is diagnosed when hemoglobin (Hb) is <13.5g/dL in males or 11.5g/dL in females, the leukocyte count is less than 4x103/μL, and the platelet count is less than 150x103/μL [1]. Pancytopenia can occur due to many hematological as well as non-hematological diseases, including exposure to toxins, radiation, or suppression of normal marrow growth and differentiation [2]. Similarly, it can result from defective hematopoiesis with cell death in the marrow, destruction of cells by the action of antibodies, or sequestration by an overactive reticuloendothelial system [3]. Bone marrow aspiration plays a very crucial role in the diagnosis and management. Bone marrow aspirate composition and cellularity vary according to the etiology. In cases of primary production defects, the marrow is generally hypocellular. On the other hand, in pancytopenia due to peripheral destruction of cells, the marrow is hypercellular or normocellular [3].

Pancytopenia is a relatively common condition with an exhaustive list of differential diagnoses, but there is relatively less discussion on this condition in developing countries, which face a huge burden of infectious diseases [4]. This study was conducted to identify the causes of pancytopenia, determine the incidence with respect to age and sex, examine the clinical presentations, and compare our findings with studies done in other parts of the world.

## Materials and methods

Study settings

The study was conducted at Hayatabad Medical Complex, a 1280-bed tertiary care hospital in Peshawar, Pakistan, which receives a large influx of patients from the whole province. The patients were admitted to the general medicine ward, gastroenterology ward, and endocrinology ward.

Study design and data collection

We conducted a cross-sectional descriptive study over seven months, from January 2, 2024, to July 30, 2024. Prior to the study, ethical approval was taken from the Institutional Research and Ethical Board (IREB) of Hayatabad Medical Complex with the approval number of '1983' (Appendix 1). A total of 106 patients were enrolled in this study who were diagnosed with pancytopenia, selected through consecutive non-probability sampling. A written informed consent was obtained from the patients to be included in the study. The patients were selected using the criteria set by De Gruchy, as follows: Hb <13.5g/dL for males and <11.5g/dL for females, total leukocyte count <4x103/μL, and platelet count <150x103/μL [5]. The absolute neutrophil count (ANC) in neutropenic patients was categorized into mild (1001-1500), moderate (501-1000), and severe (less than 500). Patients who did not consent to be included and those who were on chemotherapy or radiotherapy were excluded from the study.

Firstly, a detailed medical history was taken from the patients, including sociodemographic, drug history, smoking history, intravenous drug use, and alcohol intake. Past medical history, previous hospitalizations, and emergency visits were also taken into account. Symptoms on initial presentations, such as symptoms of anemia (pallor, fatigue, dizziness, palpitations), were recorded. Patients were also assessed for signs and symptoms of thrombocytopenia, such as bruising, bleeding, and petechiae. Patients suspected of infections were evaluated clinically and through laboratory investigations, including blood cultures and specific diagnostic tests. A thorough physical examination was done on all patients.

Subsequently, laboratory tests were conducted. A basic laboratory investigation panel was done for all patients, which included a complete blood count, liver function tests, kidney function tests, urine routine examination, and virology studies. This was followed by a series of additional blood tests on a case-to-case basis, consisting of a blood smear, blood cultures, malarial parasite (MP) test, dengue IgM, IgG, NS1 antigen, vitamin B12, folate, and thyroid function tests. According to the indications, in 50 patients, a bone marrow biopsy (aspirate + trephine) was performed and the results were recorded.

Statistical analysis

All the collected data were recorded in predesigned proforma, which were then entered into and analyzed using IBM SPSS Statistics for Windows, Version 29 (Released 2023; IBM Corp., Armonk, New York, United States). The data were analyzed across four domains: demographics, clinical presentations, laboratory findings, and etiologies. Data were outlined in the form of frequencies, means, and standard deviations and represented in graphs and tables. To determine the associations between various variables, a chi-square test was used. A p-value of less than 0.05 was considered statistically significant.

## Results

**Figure 1:**
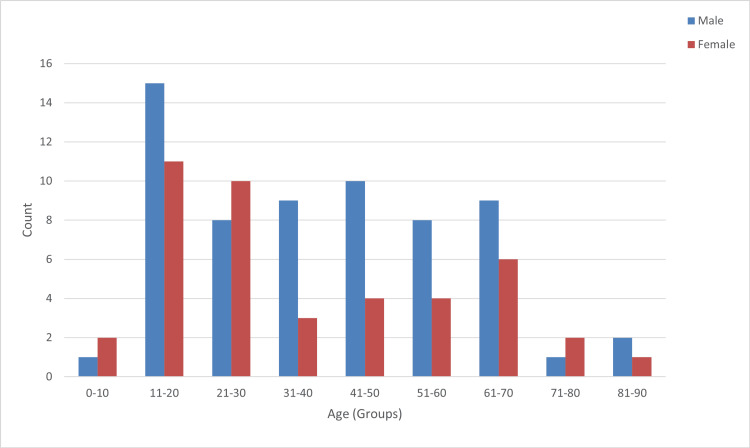
Age and gender distribution of patients

In this study, 106 cases were included, of which 63 (59.4%) were males and 43 (40.6%) were females. Male predominance was observed with a male-to-female ratio of 1.4:1. The age of the cases ranged from three years to 90 years. The mean age at diagnosis was 39 years. The majority of the patients, i.e., 44 cases (41.5%), were in the age group of 11-30 years (Figure 1).

Out of the 106 patients, 82 cases (77.3%) had no co-morbid conditions. On the other hand, nine patients (8.4%) had both hypertension and diabetes, followed by four patients (3.7%) having only hypertension. Other significant co-morbidities included tuberculosis and chronic kidney disease.

**Figure 2:**
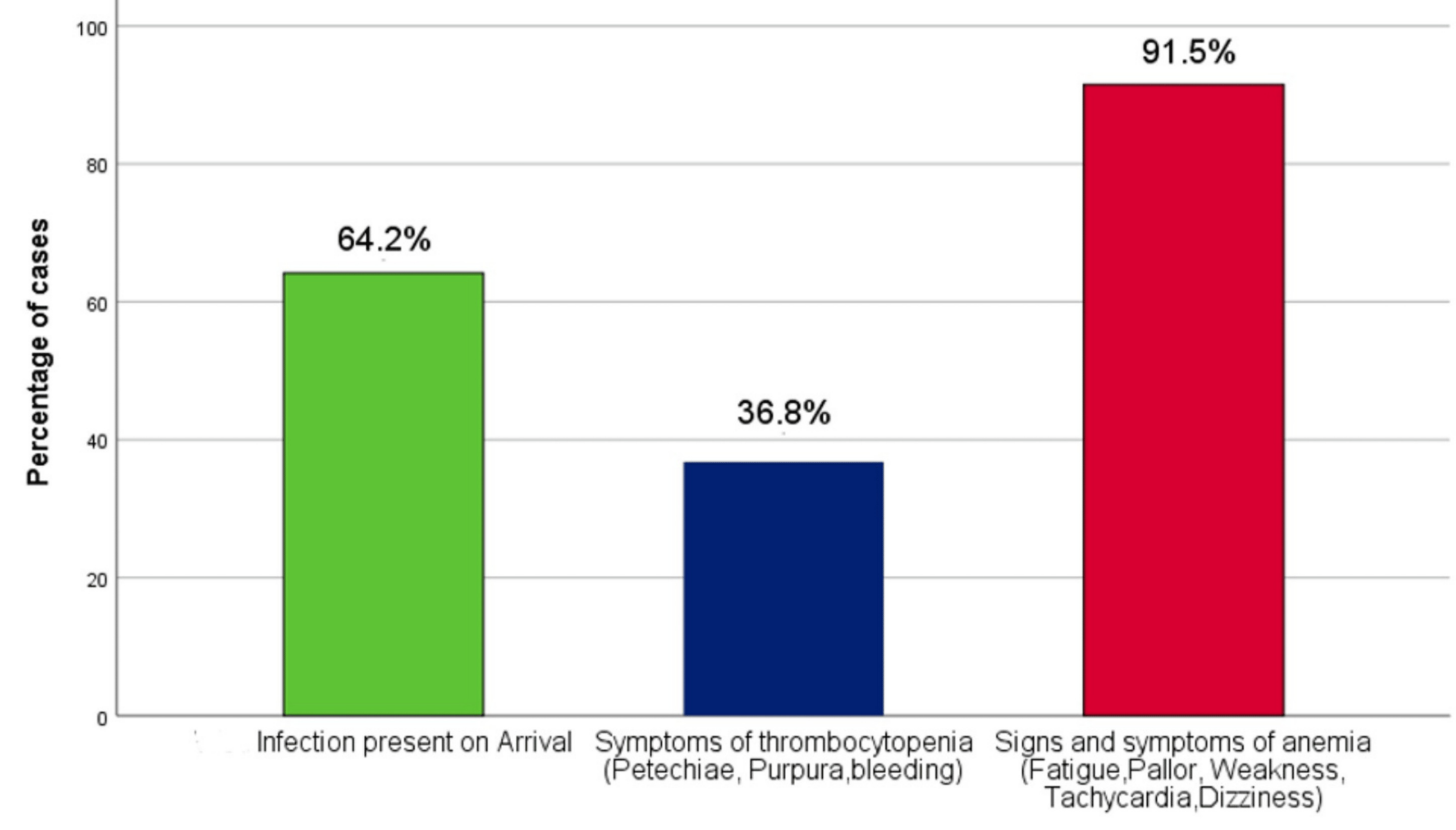
Detailed graphical representation of signs and symptoms of pancytopenia at arrival

The most commonly presenting signs and symptoms were consistent with anemia at 91.5% of the total, followed by infection and thrombocytopenia at 64.2% and 36.8%, respectively. Among infections, the commonest presentation was isolated fever (64.7%). Other significant presentations included gastrointestinal infection (17.6%), respiratory infections (7.3%), and urinary tract infections (7.3%). In patients who had signs of thrombocytopenia, the most common presentation was bleeding (58.9%). This was followed by petechiae/purpura/bruises (20.5%). The remaining cases (20.6%) had both findings (Figure 2).

Out of 106 cases, 41 had blood cultures done. Among them, 32 cultures revealed no growth. Salmonella typhi was observed in five cases, and one showed growth of Staphylococcus epidermidis.

A total of 37.7% of the cases had emergency visits to the hospital due to complications of cytopenias (infections, bleeds) in the past year. Among these patients, the commonest presentation in the emergency unit was fever (55%), followed by bleeding (45%).

**Figure 3:**
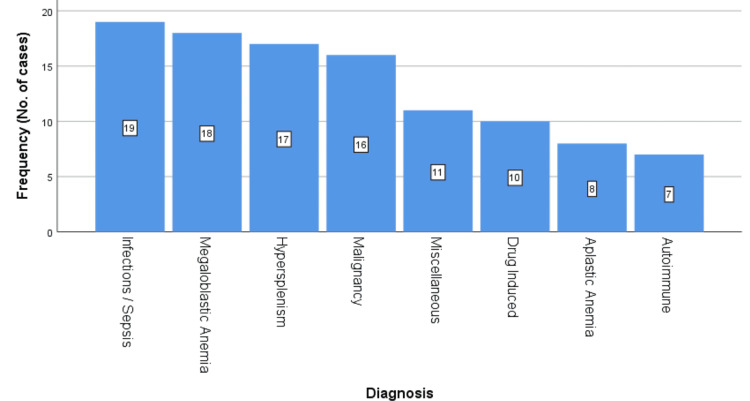
Causes of pancytopenia

Among all the causative factors of pancytopenia, infections were the leading etiology (17.9%), followed by megaloblastic anemia (17.0%), hypersplenism (16.0%), and malignancy (15.1%). Other significant causes included drug-induced (9.4%), aplastic anemia (7.5%), autoimmune (6.6%), and miscellaneous (10.4%) (Figure 3) (Table 1).

**Table 1:** Detailed description of the etiologies of pancytopenia DCLD: decompensated chronic liver disease; SLE: systemic lupus erythematosus

Diagnosis	Frequency	Percentage
Infections/Sepsis	19	17.9
Typhoid	7	6.6
Malaria	5	4.7
Tuberculosis	4	3.7
Others	3	2.8
Megaloblastic anemia	18	17
Hypersplenism	17	16
DCLD	10	9.4
Congenital hepatic fibrosis	1	0.9
Thalassemia	1	0.9
Others	5	4.7
Malignancy	16	15.1
Leukemia	9	8.4
Myelofibrosis	4	3.7
Lymphoma	2	1.8
Multiple myeloma	1	0.9
Drug-induced	10	9.4
Aplastic anemia	8	7.5
Autoimmune	7	6.6
SLE	4	3.7
Hypothyroidism	2	1.8
Autoimmune hemolytic anemia	1	0.9
Miscellaneous	11	10.4
Idiopathic	9	8.4
Hemophagocytic lymphohistiocytosis	1	0.9
Ulcerative colitis	1	0.9
Total	106	100

In infections, the most prevalent etiology of pancytopenia was enteric fever, present in 6.6% of the cases. Decompensated chronic liver disease (DCLD) was the most common cause of hypersplenism with 9.4%. Leukemia was the most common malignant etiology with 8.4% (Table 1).

A total of 57 cases had abnormal findings in blood smears, while the remaining 49 had either no abnormality seen or records could not be fetched. In those with abnormal blood smears, the most frequently observed individual finding was anisocytosis (66.6%), followed by microcytosis (52.6%), target cells (28%), and macrocytosis (21%). Overall, the most common finding case-wise was a combination of anisocytosis, microcytosis, and target cells in 14 different cases (24.5%) and isolated anisocytosis in 10 cases (17.5%). A dimorphic picture of microcytosis and macrocytosis was noted in six cases (10.5%).

A bone marrow examination was done in selected cases. In 50 patients who had a bone marrow biopsy (trephine + aspirate), the most common finding was suggestive of malignancy (32%) in which the bone marrow showed predominantly hypercellularity with abnormal cells. This was followed by megaloblastic anemia (24%) with a predominant picture of either hypercellular or normocellular marrow. Aplastic anemia (16%) showed a picture of hypocellular marrow (Table 2).

**Table 2:** Bone marrow biopsy findings

Diagnosis	Bone marrow findings	%	N
Malignancy	Hypercellular with abnormal cells	32	16
Megaloblastic anemia	Hypercellular or normocellular with megaloblastosis	24	12
Aplastic anemia	Hypocellular	16	8

In cases where pancytopenia was caused by aplastic anemia, hypersplenism, or malignancy, the reticulocyte count was <1.5% in the majority of cases. On the other hand, in megaloblastic anemia and infections/sepsis, the cases were almost evenly distributed between those with <1.5% and those with >1.5% reticulocyte count. A chi-square test was applied, and the results were statistically significant with a p-value of 0.017 (Table 3).

**Table 3:** Reticulocyte count in various causes of pancytopenia The chi-square test was applied with p-value = 0.017 (a p-value less than 0.05 was considered significant)

Reticulocyte count	Diagnosis							
	Infection/sepsis	Megaloblastic anemia	Drug-induced	Aplastic anemia	Hypersplenism	Malignancy	Auto-immune	Miscellaneous
Not done	8	2	7	2	8	9	3	6
>1.5%	6	8	1	0	1	1	3	2
<1.5%	5	8	2	6	8	6	1	3

In those cases where drugs were the causative agents of pancytopenia, the drugs involved were cyclophosphamide, azathioprine, valproate, methotrexate, sorafenib, interferons, and tenofovir.

In virology studies, hepatitis C (HCV) was more common than hepatitis B (HBV) and human immunodeficiency virus (HIV). Among cases who were positive for HCV (17), the most common diagnosis (58.8%) was hypersplenism (DCLD secondary to HCV). Both HIV and HBV were present in four cases each.

The blood counts were analyzed with the four major etiologies of pancytopenia. It was observed that infective causes were associated with the highest mean counts with hemoglobin of 8.86 mg/dL, total leukocyte count of 2.74x103/μL, and platelet count of 73.26x103/μL.

On the other hand, it was noted that cases of megaloblastic anemia had the lowest mean hemoglobin of 5.86 mg/dL, whereas malignancy had the lowest mean total leukocyte count of 2.05x103/μL as well as the lowest mean platelet count of 37.36x103/μL (Table 4).

**Table 4:** Blood counts in major diagnoses

Diagnosis (major categories)	Hemoglobin level mean ± SD (mg/dL)	Total leukocyte count mean ± SD (10^3^/μL)	Platelet count mean ± SD (10^3^/μL)
Infections/sepsis	8.86 ± 1.51	2.74 ± 0.96	73.26 ± 37.88
Megaloblastic anemia	5.86 ± 1.53	2.47 ± 1.00	57.94 ± 37.80
Hypersplenism	7.70 ± 2.05	2.44 ± 0.90	68.94 ± 37.45
Malignancy	7.76 ± 1.11	2.05 ± 1.09	37.36 ± 23.95

The presence or absence of signs and symptoms of thrombocytopenia was analyzed against the thrombocytopenia counts. The majority of the patients (64.7%) did not exhibit signs and symptoms of thrombocytopenia. Furthermore, it was observed that 21.9% presented with bleeding, while 7.6% presented with petechiae, bruises, or purpura, and 5.7% presented with both. A statistically significant number of cases who had signs of thrombocytopenia were having platelet counts of less than 50,000 (p-value = 0.001 using the chi-square test) (Table 5).

**Table 5:** Signs and symptoms of thrombocytopenia against platelet counts The chi-square test was applied with p-value = 0.001 (a p-value less than 0.05 was considered significant)

Signs and symptoms of thrombocytopenia	Platelet count (10^3^/μL)						
	101-150	51-100	31-50	16-30	0-15	Total	Percentage
No signs and symptoms	16	31	14	5	2	68	64.7%
Petechiae, bruises, purpura	0	1	0	4	3	8	7.6%
Bleeding	1	6	9	2	5	23	21.9%
Bleeding + petechiae‎/bruises‎/purpura	1	1	1	0	3	6	5.7%

The absolute neutrophil count (ANC) in neutropenic patients was compared with patients who presented with fever. Fever was present in 71.4% of the patients with ANC below 500x103/μL but only in 47.8% of those with ANC in the range 500-1000 x103/μL, and likewise, only in 47.3% of patients with ANC in the range 1001-1500x103/μL. However, a statistically significant correlation was not found after applying the chi-square test (p-value = 0.077) (Table 6).

**Table 6:** Fever in neutropenic patients The chi-square test was applied with p-value = 0.077 (a p-value less than 0.05 was considered significant)

Absolute neutrophil count (x10^3^/L)	Patients with fever N (%)	Patients without fever N (%)
Severe (0-500)	15 (71.4%)	6 (28.5%)
Moderate (501-1000)	11 (47.8%)	12 (52.1%)
Mild (1001-1500)	9 (47.3%)	10 (52.6%)

## Discussion

Pancytopenia, a common hematological finding, can cause multiple diseases. The underlying cause varies depending on age, ethnicity, geographical distribution, and age. The prognosis and treatment of pancytopenia depend on early diagnosis, etiology, and comorbidities associated with it. About 106 cases of pancytopenia were studied to further add to the knowledge in this regard.

The male-to-female ratio in the study was found to be 1.4:1, with male predominance. This is concurrent with the findings of Hagler et al. showing a male-to-female ratio of 1.2:1 [6]. Similarly, studies carried out at Isra University Hospital (Hyderabad) [7], Bolan Medical Complex Hospital (Quetta) [8], and Calcutta National Medical College and Hospital [9] reported it to be 1.8:1, 2:1, and 1.2:1, respectively. However, a study carried out at Lady Reading Hospital showed females constituted about 62% of the affected population [10]. Another study conducted at a tertiary care hospital in Karachi reported equal distribution among the genders [11]. Hence, it shows that male predominance is usually reported, but these variations are subject to different variables such as locality, age, etc.

The median age of the patients in our study was found to be 39 years. This is in agreement with Samreen Z et al. [8] and Farooque R et al. [11].

In this study, we found that the most common clinical presentation was consistent with anemia (91.5%), followed by signs and symptoms of infection (64.2%) and thrombocytopenia (36.8%), respectively. Similar to the findings of the study carried out by Gajbhiye SS et al. [9] with signs and symptoms of anemia (78%), infection (68%), and thrombocytopenia (56%). This, however, is in contrast to studies carried out by Alim M et al. in children, who reported fever to be the most common presenting symptom at 78.6%, followed by pallor at 70% [12]. Thus, variations may exist in the presentation depending on different factors such as age, ethnicity, etc.

Among the clinical features of thrombocytopenia, the most common presentation was bleeding (58.9%), followed by rash (20.5%), whereas the remaining had mixed features (20.6%). This was also reflected in the study carried out by Gajbhiye SS et al., who reported bleeding and rash at 57% and 42%, respectively [9].

Pancytopenia can be caused by many underlying conditions, and the frequency varies depending on different factors such as age, gender, and geographical area. 

Being a developing country, infection (17.9%) was found to be the leading cause of pancytopenia. Whereas, megaloblastic anemia was found to be the next frequent cause (17%). Among the infections, typhoid and malaria were found to be the major contributors, making up about 63% of the total cases. Megaloblastic anemia, however, was due to nutritional deficiency. Thus, infection and megaloblastic anemia are the prime contributors to pancytopenia.

This is in congruence with studies by Hamid GA et al. [13] and Tareen SM et al. [14], where infection (malaria) was found to be the most common cause at 30.6% and 29.44%, respectively. Additionally, studies conducted by Ishtiaq O et al. [15], Aziz T et al. [16], and Farooque R et al. [11] reported megaloblastic anemia to be the leading cause of pancytopenia, accounting for 39%, 49%, and 41.7%, respectively. Similar results were also concluded from studies in India by Gore CR et al. [17] and Das MK et al. [1]. Hence, reversible causes make up the major element of pancytopenia.

Hypersplenism made up 16% of cases of pancytopenia. DCLD accounted for about 9.4% of cases, with HCV as a major contributor and HBV and HIV as minor contributors. This is in agreement with the high burden of the disease reflected in an article by Asif AF et al. [18]. Studies by Hamid GA et al., Farooque R et al., and Hayat AS et al. observed similar findings of hypersplenism contributing about 20%, 16.7%, and 15.3%, respectively [19].

Though malignant causes of pancytopenia are predominant in the West, it cannot be overlooked in our study as it accounted for 15.1% of the total cases. With leukemia predominating at 8.4%, other malignancies included myelofibrosis, lymphoma, and multiple myeloma. Similarly, studies by Zeeshan R et al. [20] reported leukemia at 13.9% and Zeb JA et al. [21] reported leukemia at 23.9% in the pediatric population with pancytopenia, which shows that leukemia is much more common in the pediatric population in developing countries. The rising trend can be attributed to urbanization, chemical, and radiation exposures.

Drug-induced pancytopenia made up 9.4% of the total cases studied. While many drugs are known to cause pancytopenia, our study concluded cyclophosphamide, azathioprine, valproate, methotrexate, sorafenib, interferons, and tenofovir to be the main agents.

Among the minor etiologies, aplastic anemia was found to be the culprit in 7.5% of cases. That being said, the contribution of aplastic anemia varies from region to region, e.g., a study conducted in Rawalpindi [20] showed only 5.2% of cases caused by it; however, a study from North India [12] reported about 32.1% cases, while one in Yemen [13] reported 10% cases caused by it. Autoimmune diseases such as systemic lupus erythematosus systemic lupus erythematosus (SLE), hypothyroidism, and autoimmune hemolytic anemia (AIHA) made up about 6.6% of the cases, which is comparable to the 6.2% reported by Zubair AB et al. [22]. Similarly, Farooque R et al. [11] and Khan MI et al. [23] reported 12.5% and 1.3%, respectively.

Miscellaneous causes of pancytopenia were represented by a small percentage (10.4%), with idiopathic dominating at 8.4%. Most of these idiopathic cases improved during admission, where they were started on supportive treatment and the exact cause couldn’t be identified. However, bearing in mind these patients were also started on antibiotics, infectious causes among these patients couldn’t be ruled out.

In this study, we found that only 57 patients had findings on blood smears. Others had either no findings or blood smears could not be found due to several limitations. However, with the limited information we had, we concluded anisocytosis to be the predominant finding, followed by microcytosis and macrocytosis. However, these findings were presented in combination rather than isolated findings. A combination of anisocytosis, macrocytosis, and microcytosis was found to be the most common among the cases (24.5%). Isolated anisocytosis and a dimorphic picture were reported at 17.5% and 10.5%, respectively. This is in contrast to the study of Gayathri BN et al. [24], who reported dimorphic anemia to be the predominant finding at 37.5%, followed by macrocytic anemia at 31.7%.

A total of 50 cases had bone marrow biopsy, where hypercellularity with abnormal cells was the most common finding keeping up with malignancy. This was followed by hypercellular or normocellular bone marrow in cases of macrocytic anemia and hypocellular bone marrow in cases of aplastic anemia. Gayathri BN et al. [24] also reported similar findings in cases of malignancy and aplastic anemia.

Fever was common in neutropenic patients, particularly in severe neutropenia. However, with a p-value of 0.07, a statistically significant correlation was not found. This was also concluded by Gajbhiye SS et al. in their study in India [9], thus leaving room for more studies to be carried out to get a better understanding of the matter.

The majority of the patients with thrombocytopenia did not have any bleeding tendencies, while others had bleeding, petechia, or both. After comparing the severity of thrombocytopenia against bleeding tendencies, it was found that the majority of the patients with bleeding tendencies had a platelet count of less than 50,000. This aligns with the study conducted in India, where most of the patients who had bleeding tendencies were found to have a platelet count below 25,000 [12].

Being a developing country with infections forming the bulk of our cases, blood cultures were done in 41 cases. While the culture did not grow anything in the majority of the cases, Salmonella typhi was grown in five cases. Salmonella is notorious for causing pancytopenia [25,26]. Since Salmonella is spread by contaminated food and water, addressing this issue can significantly reduce the incidence of pancytopenia, as infection was identified as the leading cause in our study.

Preventing Salmonella typhi infection requires strict adherence to safe food handling practices, access to clean water, and robust personal hygiene measures, including regular handwashing. Public health initiatives that promote sanitation and educate communities on these practices are crucial in minimizing transmission and protecting public health. Similarly, preventing megaloblastic anemia primarily involves ensuring adequate dietary intake of essential nutrients, particularly vitamin B12 and folate, through a balanced diet rich in fruits, vegetables, whole grains, and animal products. Additionally, education about the importance of supplementation for at-risk populations, such as pregnant women and individuals with malabsorption conditions, is vital to reducing the incidence of this condition.

Concluding the findings above, our study did have its limitations. Our study was done at a single tertiary care hospital, and including other hospitals would have allowed us a more comprehensive understanding of etiologies. Additionally, vitamin B12 and folate levels in patients with megaloblastic anemia couldn’t be performed as these tests were not available in the hospitals set up, and due to financial constraints, most of the patients refused to get them from private laboratories. Another limiting factor was that most of these patients were already on vitamin B12 and folate supplements, rendering the tests futile for evaluation. Nonetheless, our study concludes that addressing the nutritional and infectious causes can play a major role in the prevention of pancytopenia. Further research is encouraged looking into the risk factors and causes according to the different age groups with a larger sample size involving multiple healthcare centers.

## Conclusions

While pancytopenia results from a wide range of diseases, our research focuses primarily on the etiologies in a developing country with a huge infectious disease burden. The research showed that infections and megaloblastic anemia were the leading causes, followed closely by hypersplenism and malignancy. Among infections, typhoid was the most common culprit found. By recognizing the common etiologies and correlating them with symptoms and laboratory findings, we can promptly diagnose the cause and treat reversible conditions, thereby preventing complications and overinvestigation. The provision of empirical antibiotic treatment and nutritional supplementation can counter the two major contributors of pancytopenia.

## Figures and Tables

**Figure 4:**
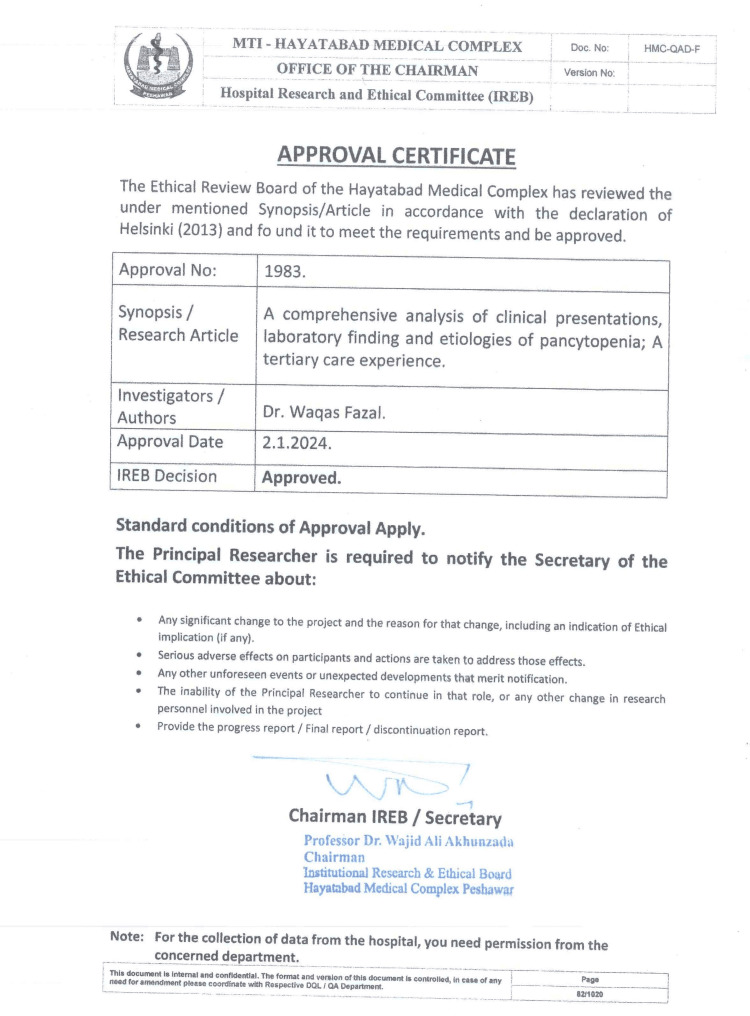
Ethical approval

## Appendices

Appendix 1 
